# Clinical Research Evidence Supporting Administration and Dosing Recommendations of Medicinal Cannabis as Analgesic in Cancer Patients

**DOI:** 10.3390/jcm12010307

**Published:** 2022-12-30

**Authors:** Catalina Christensen, Morten Allesø, Martin Rose, Claus Cornett

**Affiliations:** 1 Tetra Pharm Technologies ApS, Soendre Jernbanevej 13, DK-3400 Hilleroed, Denmark; 2 Department of Pharmacy, University of Copenhagen, Universitetsparken 2, DK-2100 Copenhagen, Denmark

**Keywords:** cancer pain, medicinal cannabis, cannabinoid-based medicine, endocannabinoid system, clinical evidence, administration, dosing

## Abstract

The analgesic potential of *Cannabis sativa* L.—based medicinal cannabis products for treatment of cancer associated chronic pains has gained increased interest in recent years. To ensure a controlled distribution of these products and investigate their therapeutic potential, several countries have established so-called pilot trials. Many doctors, however, are hesitant to prescribe medicinal cannabis primarily due to lack of research evidence regarding the products’ efficacy, safety and thus questionable dosing guidelines. This review aims to elucidate clinical research supporting administration of medicinal cannabis in cancer patients for analgesic purposes. The cannabinoids’ effects on the endocannabinoid system (ECS) and its implication in pain regulation is included to illustrate the complexity related to this research field. Published clinical studies on medicinal cannabis primarily consist of observational studies and only one pilot randomized controlled trial (RCT), where more RCTs exist on the cannabis-based product, Sativex^®^ (GW Pharma Ltd., Cambridge, UK). The studies indicate analgesic potential, however non-significantly, for most patients and with acceptable safety profile. Summarizing, high-quality RCTs are scarce in this research field, and the limitations of the observational studies complicates interpretation of clinical outcomes. Despite discrepancy among the studies, they do show indications for administration and dosing regimens providing analgesic effects for some cancer patients.

## 1. Introduction

Administration of the cannabis plant (*Cannabis sativa* L.) as an alternative to conventional medicines in the management of various diseases and disease-related symptoms, including cancer-associated pain, has increased massively during the last decades [1,2]. It has been postulated that we are currently experiencing a re-medicalization of cannabis, as more patients express interest to try out treatment with cannabis for medicinal purposes [3]. This tendency is believed to be caused by several factors, among other the discovery of the endocannabinoid system (ECS), which has accelerated the development of cannabis-based medicinal products.

Although documentation is sparse, it is generally recognized that patients suffering from various conditions have been sourcing cannabis illegally for symptom relief, thereby accepting the inherent risks of such an unregulated treatment strategy [3,4,5]. Supported by the rapidly growing understanding of the pharmacology of cannabinoids, this has led to an increased focus on and demand for legal medicinal cannabis, i.e., produced within the frameworks of Good Agricultural Collection Practice (GACP) and Good Manufacturing Practice (GMP) frameworks [6,7]. As a result, several countries have developed policies and pilot programs allowing for the prescription of cannabis for medicinal purposes [3]. Denmark’s pilot program (running from 2018 till the end of 2025) allows Danish doctors to prescribe medicinal cannabis, however only if authorized conventional medicinal products have been tried without satisfactory effects. The medicinal cannabis products have not yet been researched in-depth through high-quality clinical randomized controlled trials (RCTs), which means that evidence regarding their safety and efficacy in different patient populations is scarce and highly needed. This challenges the administration and dosing of the products in a clinical setting [8].

The term “medicinal cannabis” is applied broadly to products containing either extracts from the *Cannabis sativa* L. plant or isolated active compounds or synthetic analogues hereof. To avoid confusion and provide clarity, the product types in scope of this review are specifically defined based on the existing formal definitions as follows: The medicinal cannabis products that are part of several pilot programs currently running are defined as products containing the full spectrum of naturally occurring compounds that can be extracted from the *Cannabis sativa* L. plant, thereby referred to as full-spectrum medicinal cannabis [8]. Another product type is the cannabis-based medicines, such as the authorized medicinal product Sativex^®^, also known as nabiximols, approved for cancer-associated pain in Canada [9]. Sativex^®^ is a natural *Cannabis sativa* L. plant extract product, composed of a combination of extracts from a high cannabidiol (CBD) cultivar and a high ∆-9-tetrahydrocannabinol (THC) cultivar, combined to a final drug product composed of almost equal amounts of CBD and THC [10].The additional content of unlabeled naturally occurring compounds (e.g., residual cannabinoids and terpenoids) from extract of the *Cannabis sativa* L. plant defines Sativex^®^ as a so-called broad-spectrum product [11]. Other types of products are based on single cannabinoids, either isolated from the cannabis plant or produced through chemical synthesis. In the context of this review, these are considered active pharmaceutical ingredients (APIs) [12].

To the best of our knowledge, clinical evidence of medicinal cannabis as an analgesic in the management of cancer pain is limited both in scope and in quality, while clinical evidence of higher quality, in the form of RCTs, exists in support of the analgesic effects of the cannabis-based product Sativex^®^ in cancer patients. However, the evidence behind it is based on only a few clinical trials [1,13].

Despite the lack of high-quality research evidence, it is not uncommon to administer cannabis within clinical settings to manage chronic pains often associated with cancer [1]. Several studies confirm this tendency, with cannabis consumption observed prevalently for both symptom management, in particular pains, and for the purpose of treating the underlying disease [14,15]. It has been estimated that among 80% of advanced cancer patients, irrespective of cancer type, suffer from disease-related pains, with as many as a third of the survivors reporting to experience symptoms of pain [16]. Because of improved methods to diagnose the patients, the population of cancer patients surviving the disease is believed to increase in the future. Cancer-associated pains are highly heterogenous and complex to treat satisfactorily and completely in all patients. The pain can be caused by the cancer tissue itself or as an adverse effect of the therapeutic management of the cancer, e.g., through chemotherapy. The currently most abundant and primarily used analgesic is opioids. However, opioids often do not provide satisfactory analgesia for all patients and additionally often comes with a line of adverse effects and an increased risk of developing addiction. Thus, frequent opioid usage often requires addition of other medicinal products to manage the adverse effect symptoms [17,18].

Several challenges pertain to performing research within the medicinal cannabis field: (1) the synergistic and complex effects occurring when administering hundreds of molecules into the body all at once, (2) the molecular composition of the herbal material (i.e., either as dried biomass or extract) being affected by the cultivation and processing factors, resulting in inter-supplier product variability even when label claim of primary cannabinoids are identical, (3) the holistic multi-functionality of the ECS itself, where inter-individual and population-based differences exist, and (4) the complex heterogeneity related to cancer pain etiology. These factors, among others, add to the high complexity of medicinal cannabis research, which—combined with the current low-quality clinical evidence of its efficacy—challenges the preparation of evidence-based recommendations to support the administration and dosing guidelines in the clinical settings [19,20]. A study, investigating cancer patient survivors’ perceptions of barriers against using medicinal cannabis, reported the following reasons: lack of research evidence, concerns of dependency and healthcare providers’ criticism towards medicinal cannabis usage [21]. The hesitancy of doctors to prescribe these products, has left many patients without professional medical guidance and as a result have turned to the illegal market to source their cannabis to manage their disease [22,23]. These illegal products do not come with a GMP-compliant certificate of analysis and thus may potentially cause harm to the patient, either due to little or no efficacy or because of harmful components that were not controlled during production (e.g., erroneous cannabinoid content, foreign matter, heavy metals, pesticides, mycotoxins, microbes, etc.) [3,14]. Ultimately, this causes a societal challenge for authorities to tackle.

The rapid increase in product availability calls for evidence-based practical guidelines to support the process of decision-making by physicians. Recently, a review study was published by Gorzo et al. [24], providing an updated status of existing research evidence of relevance to oncologists in their consideration of administrating cannabis-based medicines in the management of cancer pain. The study found that while convincing pre-clinical research exist, the translation of these data into high-quality clinical trials is lacking, which is necessary to determine their clinical relevance and safety. Current selective review contributes to this field through a comprehensive summary of existing clinical research evidence supporting the analgesic effects of medicinal cannabis in cancer patients. A particular focus will be on research that potentially qualifies as a guidance to practitioners for the administration and dosing of medicinal cannabis to patients suffering from cancer-related pains. Only clinical research regarding medicinal cannabis (i.e., full-spectrum) and the, thus far only, cannabis-based product Sativex^®^ will be considered relevant for the purpose of this review. In addition, the clinical findings will be discussed in the context of challenges related to the field of medicinal cannabis research, such as the multi-faceted role of the endocannabinoid system, lack of product standardization and unavailability of a true placebo. It is important to state that this review should not be perceived as a guideline nor a recommendation for treatment with medicinal cannabis as a replacement to standard conventional treatments. It is rather to be considered as an objective, yet critical, overview of published studies that physicians and professionals may consult at their own discretion.

The review is based on a thorough selective literature search performed in the databases and platforms; PubMed^®^ (Bethesda, MD, USA) (MEDLINE), ScienceDirect^®^ (Amsterdam, The Netherlands) and Cochrane Library. Included clinical studies have been published in the period from February 2010 till September 2022. Search terms includes: “medicinal cannabis, i.e., medical cannabis”, “full-spectrum medicinal cannabis”, “cancer pain”, “malignant pain”, “endocannabinoid system” and “entourage effect”. Reference lists of selected articles have been reviewed for additional relevant articles.

## 2. The Endocannabinoid System and Its Implication in Cancer Pain

The ECS is an endogenous multifunctional pro-homeostatic signaling system being almost ubiquitously distributed within the body. The system is generally recognized to consist of three main parts (1) receptors: G protein-coupled receptors (GPCRs); cannabinoid receptor 1 (CB1R) and 2 (CB2R), (2) endocannabinoids: the body’s own signaling molecules regulating the ECS through the cannabinoid receptors, including N-arachidonylethanolamine (anandamide, i.e., AEA) and 2-arachidonoylglycerol (2-AG) and (3) the enzymes: responsible for the metabolism and regulation of endocannabinoids available at a given time. In line with the development of research in the field, additional components have been discovered being part of the ECS. This including (1) receptors: GPCRs (e.g., GPR18, GPR55 and GPR119), ion channels (e.g., Transient Receptor Potential Vanilloid 1 (TRPV1) and nuclear receptors (e.g., Peroxisome Proliferator-activated receptor gamma (PPAR-y), (2) endocannabinoid-like compounds: e.g., Palmitoylethanolamide (PEA) and Oleoylethanolamine (OEA) and (3) synthesizing and degradative enzymes and transport proteins of the endocannabinoids and alike ligands. The ECS acts locally at the various body parts it is localized within, where it responds to and is activated by disturbances occurring within these systems, with the aim to recover and maintain homeostasis [25,26].

**Figure 1:**
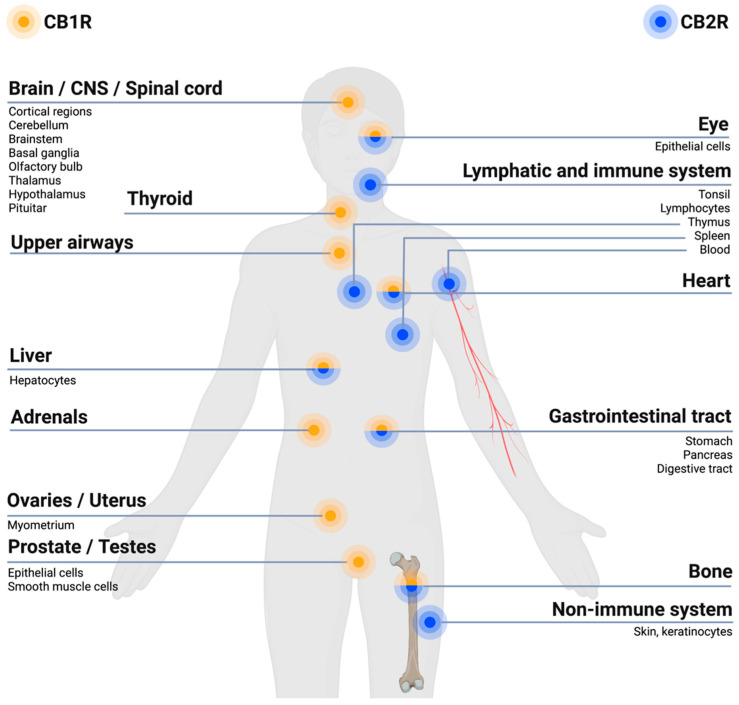
Endocannabinoid system (ECS) receptor distribution in the body. In general, 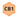 receptors (CB1Rs) are primarily expressed within brain regions and the nervous system, especially in the central nervous system (CNS), and to a lesser extent at other sites of the body. 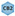 receptors (CB2Rs) are primarily expressed within cells related to the immune system and peripheral body tissues [27,28]. Created with BioRender.com.

The ECS has receptors located at various body sites, such as in the central nervous system (CNS), peripheral nervous system (PNS), immune system cells and peripheral tissues, see Figure 1.

**Figure 2:**
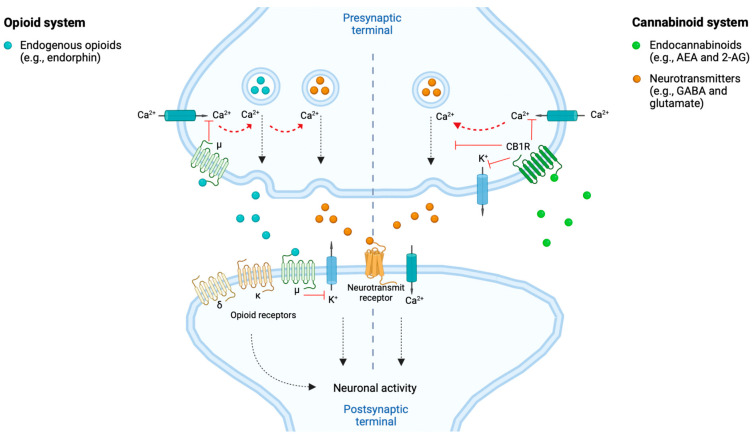
The opioid- and cannabinoid systems implicated in the modulation of neuronal activity and pain signal. Both endogenous opioids (e.g., endorphins) and endocannabinoids (e.g., anandamide, i.e., AEA and 2-arachidonoylglycerol, i.e., 2-AG) are involved in the response to and modulation of pain signaling. This through reducing the neuronal activity and thereby pain signaling. The endogenous opioids can, e.g., act at the postsynaptic neurons’ opioid receptors (i.e., δ, κ and μ) and at the presynaptic neurons’ μ receptors. The endocannabinoids can, e.g., act at the presynaptic neurons’ cannabinoid 1 receptors (CB1Rs). Both pathways, when activated by their respective agonists, modulate the intracellular release of neurotransmitters (e.g., GABA and glutamate) through calcium (Ca^2+^)-dependent vesicular release blockade, by blocking Ca^2+^ and kalium (K^+^) channels. Hereby is the neuronal activity decreased and results in dampening of the pain signaling [28,33,34]. Created with BioRender.com.

The ECS is involved in regulating pain within the body due to its expressions of cannabinoid receptors within the different pain pathways involved in the transmission of pain signals throughout the body. Overall, the ECS exerts an inhibitory effect on pain signaling [29]. The CB1Rs and CB2Rs are differently located within the body, with the CB1Rs predominantly localized within brain regions and the nervous system, in particular the CNS, while CB2Rs are predominantly found within cells of the immune system and the PNS, as illustrated in Figure 1 [28,30]. The CB1Rs have been found particularly at presynaptic sites of neurons within the CNS. Upon activation of the CB1R an inhibition of neurotransmitter release occurs, resulting in a dampening of the pain signaling [30], as illustrated in Figure 2. Upon activation of the CB2R, the transduction of pain signaling from the PNS into the CNS is inhibited thus reducing, among other, nociception (pain sensation) and inflammation [29,30], qua., the holistic function of the ECS. A deficiency of endocannabinoids thereby causes a hypofunctional ECS and has been proposed to be linked to a decreased pain threshold because of homeostatic ECS functions that cannot be maintained [31].

The ECS regulates and activates the cannabinoid receptors through the endocannabinoids, where especially the functioning of AEA and 2-AG has been studied and discussed within the research literature [32]. Upon binding of the endocannabinoids to the CB1R, an intracellular signaling cascade is initiated, which ultimately inhibits neurotransmitter release, thereby blocking pain signaling (Figure 2) [32,33]. AEA has been observed to bind as a partial agonist at the CB1R and CB2R, with a higher affinity at the CB1R, where 2-AG has been observed to be an agonist of both the CB1R and CB2R, binding with low to moderate affinity [28,32,34].

Cannabinoid receptors share several characteristics with the opioid receptors, such as their potential to induce analgesic effects through their neuronal presynaptic expressions within neurons whereby they inhibit neurotransmitter release and decrease the neuronal activity and thus, e.g., pain signaling [1,33], as illustrated in Figure 2. Both receptors belong to the GPCR family of the subtype Gi/o, thereby sharing intracellular signaling mechanisms upon stimulation by their respective ligands, such as reduction in activity of calcium voltage-dependent channels [35]. It has been proposed, based on pre-clinical studies, that CB1Rs and μ opioid receptors can interact by forming heterodimers in neurons where they are co-expressed [36]. Furthermore, cannabinoids have been proposed to stimulate synthesis and release of endogenous opioids and vice versa [37]. These characteristics overall support the occurrence of interactions among opioids and cannabinoids in inducing synergistic additive analgesic effects when co-consumed ([2], pp. 2–4, [35]).

## 3. *Cannabis sativa* L. Compounds Possessing Analgesic Effects

More than 100 different (phyto)cannabinoids have been discovered among the existing cultivars of *Cannabis sativa* L. plants. Only some of them have been investigated in-depth, particularly THC and CBD. The cannabinoids possess different receptor affinities at different targets within the body, both within and outside the ECS, explaining partly the diverse therapeutic potential of different cannabis cultivars [38].

Common cannabinoids such as THC, CBD and cannabinol (CBN) possess affinities for the cannabinoid receptors and thereby to some extent mimic the binding of endocannabinoids [30]. CBN binds to and modulates the cannabinoid receptors, with highest affinity at the CB2R and weak agonistic binding at the CB1R [30,38]. THC shows partial agonistic as well as antagonistic binding to both the CB1R and CB2R, depending on factors such as the concentration of THC consumed, cell type specific receptor expressions and presence of other cannabinoids (i.e., endocannabinoids or exocannabinoids, the latter derived from outside the body) [30]. CBD possesses low affinities and no agonistic binding at the CB1Rs and CB2Rs but is on the contrary believed to exert negative allosteric or antagonistic binding at the CB1R [32,39]. It is suggested that CBD can positively modulate THC-mediated mechanism of actions and thus reduce the adverse psychoactive effects related hereto, which allows for higher doses of THC to be administered and an overall increased therapeutical effect [40]. This might aid in explaining the therapeutic benefits of co-administering CBD and THC to cancer pain patients, e.g., as, demonstrated for Sativex^®^ in a clinical study [41]. In addition, CBD indirectly enhances the level of AEA through inhibition of its cellular re-uptake [30]. Another GPCR, the GPR55, was found to be activated by THC, CBD and 2-AG, with THC and 2-AG possessing higher agonistic efficacy and potency, respectively, for this receptor over CB1R and CB2R. Thus, the GPR55 has been proposed to in fact be the third cannabinoid receptor [42]. Minor cannabinoids such as cannabigerol (CBG) and cannabichromene (CBC) have been demonstrated to exert analgesic effects through affecting other targets than the cannabinoid receptors (e.g., inhibition of AEA re-uptake, inhibition of certain serotonin receptors, inhibition of enzymes involved in synthesis of inflammatory mediators and activation of certain adrenergic receptors) [30]. The ability of minor cannabinoids to interact with targets within and outside the ECS has led to the proposal of an expanded endocannabinoid system, or the so-called endocannabidiome [43,44], which is believed to further expand as more research is conducted.

Terpenoids are another group of compounds contained within the cannabis plant. They are responsible for the characteristic aroma associated with cannabis and believed to possess individual bioactive effects in addition to modulatory effects of cannabinoids, however to the best of our knowledge currently mainly based on pre-clinical research. More than 200 different terpenoids have been detected within the *Cannabis sativa* L. plants, while only the terpenoids β-caryophyllene, β-myrcene and D-limonene have been observed to exert analgesic effects [45]. Much about their mechanism of actions is still unknown, although pre-clinical studies of β-caryophyllene have demonstrated agonistic binding to the CB2R, particularly the peripherally located, thereby exerting synergistic effects with THC [30].

A Pharmacological Perspective on the Entourage Effect Mechanism Theory

The entourage effect mechanism theory has been proposed to be the underlying reason for many patients claiming to experience an overall better effect when consuming full-spectrum cannabis products compared to products containing single cannabinoids [46]. Research aims to establish the pharmacological effects that collectively contribute to the entourage effect as it is currently not supported by solid research evidence [47]. The mechanism of actions are still unknown but are in general believed to be caused by pharmacokinetic as well as pharmacodynamic interactions between the bioactive compounds consumed from cannabis products. Pharmacokinetic interactions affect the absorption, distribution, metabolism, and excretion of the other compounds. This ultimately impacts the bioavailability, while pharmacodynamic interactions affect the efficacy of the bioactive compounds, by targeting receptors or enzymes and thereby enhancing or suppressing the bioactive effects of other compounds. However, both synergistic, additive and antagonistic effects among the compounds of *Cannabis sativa* L. have been demonstrated [48]. Different mechanisms have been proposed to cause this overall synergistic effect, including effects on the pharmacological properties of the individual compounds, which ultimately affects the clinical efficacy and occurrence of potential adverse effects [45].

Terpenoids have been proposed to act as entourage compounds by for example acting as vasodilators of the alveolar capillaries, thereby increasing the permeability of the blood–brain barrier, whereby the pharmacokinetic effects related to, e.g., THC is increased [49,50]. β-myrcene specifically has been found to possess multiple mechanism of actions, e.g., enhancing CBD’s effects on hepatic cytochrome P450 (CYP) enzymes. CBD has been reported to inhibit the subtype CYP2C9, which is responsible for the metabolism of THC into its more psychoactive breakdown product, 11-OH-THC [30,51]. This potentially contributes to enhance the beneficial effects of THC and CBD while reducing the adverse effects associated with the psychoactivity of THC and its breakdown product. However, this can potentially also affect the metabolism of additional co-consumed medicinal products metabolized by the same hepatic enzymes, whereby the pharmaceutical effects can be increased or decreased, ultimately affecting the clinical outcome [30]. As CBD exerts multiple effects that affect the bioactivity of THC, it has been proposed that CBD is an entourage compound. These findings have led to the proposal of THC being perceived as a “silver bullet” and the additional bioactive cannabis compounds and the cannabis plant itself as a “synergistic shotgun”, with the entourage effect been referred to as the cannabis plant synergy [45,46].

In summary, the ability of cannabinoids to bind to a multitude of receptors, potentiate or even block the effects of one another adds to the complexity of medicinal cannabis research. For full-spectrum products, the presence of additional potentially bioactive compounds, like terpenoids, furthermore adds to the complexity and challenges the production of a truly standardized product for clinical testing, ultimately reducing the statistical power of a clinical study design. The presented complexity of the ECS and the compounds of the *Cannabis sativa* L. plant should be kept in mind when critically assessing the outcomes of clinical trials investigating full-spectrum medicinal cannabis products for relief of cancer-induced pain.

## 4. Existing Clinical Research Evidence

Clinical studies assessing the analgesic effects of full-spectrum product types are mostly observational and not performed according to a RCT design, despite one pilot RCT recently published [52]. The main clinical study characteristics and outcomes are summarized in Table A1.

### 4.1. Clinical Studies with Medicinal Cannabis (Full-Spectrum)

A prospective study [53] analyzed the efficacy and safety related to medicinal cannabis consumption in a large cancer patient population (N = 2970). Pain was stated as being the main cause for consumption (77.7%). At the start of treatment more than half (52.9%) of the cancer pain patients reported very high pain intensities, i.e., 8–10 on a visual analogue scale (VAS) ranging from 0–10 (0 = no pain, 10 = worst pain possible), with only 4.6% stating this after six-month treatment (*p* < 0.001). 16 different cannabis cultivars were available to the patients to choose from, where most patients consumed different variants and a vast majority (91.8%) preferred THC-rich cultivars of the indica subtype. The authors concluded that treatment with medicinal cannabis in the palliative care of cancer patients is effective, safe, and well tolerated. Based on clinical data derived from the same database, the same research team recently published another prospective study [54]. This study additionally supported the safety and efficacy related to medicinal cannabis consumption for 6 months across a large patient population, including cancer patients (N= 4.205), of which 76% reported pain (N = 3173). Different cannabis cultivar consumption patterns were observed across disease populations, with high-THC cultivars in general being preferred, as illustrated in their previous study. A tendency, similar to what was concluded from their former 2018 study, of significant pain intensity reduction (*p* < 0.001) was established after medicinal cannabis consumption over a 6-month period. Another prospective study [55] investigated the effect of short-term one-month medicinal cannabis treatment in cancer patients undergoing palliative care. As with the former studies, different cannabis cultivar products were available to the patients (THC- or CBD-rich or THC:CBD). Overall, all the different cultivars were observed to significantly (*p* < 0.05) reduce pain intensity. Based on a numerical pain scale (NPS) ranging from 0–10 (0 = no pain, 10 = most intense pain imaginable), weekly average pain intensity improved from 7(3–8.8) to 5(1.5–7), with CBD-rich cultivars found equally effective as THC-rich. The investigators cautiously concluded recommended CBD-rich cultivar prescriptions over THC:CBD or THC-rich.

A retrospective study [56] investigated the role of medicinal cannabis in cancer pain patients (N = 232). Patients consuming cannabis reduced their pain score meanwhile reducing co-consumption of opioids. It was concluded that cannabis consumption may be considered as adjuvant analgesic treatment in the palliative care pain management of cancer patients. In line with this, Zarrabi et al. [57] presented seriously ill patients (N = 101), of which 76% were advanced cancer patients of an outpatient palliative care practice, to an online survey investigating their perceptions towards medicinal cannabis. 96% of the patients stated that cannabis was helpful in the management of their pains and additionally improved their feeling of wellbeing and 62.2% of the cancer patients stated cannabis being important for their cancer cure. Sura et al. [58] found that out of 93 cancer patients receiving medicinal cannabis through prescription at a palliative care center, 48% reported improvement in pain while 45% reduced their opioid intake. Few non-serious adverse effects were reported and 50% consumed cannabis for its anti-cancer curative properties. A prospective long-term study [59], including a 6-month follow-up, was published, investigating safety and efficacy related to medicinal cannabis consumption in the management of cancer symptoms, including pain. It was observed that long-term medicinal cannabis consumption induced mild to modest statistical improvements in cancer-associated symptoms, including pain. Positive change (i.e., pain decrease) in average pain intensity from baseline till 6-month follow-up was reported by 36%, who experienced more than a 30% pain reduction. A significant (*p* < 0.001) 20% reduction in average weekly pain intensity (median 7(3–9) to 5(3–7)) was observed. It was concluded that medicinal cannabis can be perceived as a safe treatment option associated with no serious adverse effects.

A cohort study (N = 16) from the United Kingdom (UK) was published recently. The study aimed to elucidate experiences and outcomes related to cannabis-based medicinal product prescriptions in palliative care settings, including cancer pain patients [60]. These preliminary data showed that cannabis-based medicinal products were well-tolerated and associated with few adverse effects, being mild to moderate. Pain was reduced from severe (VAS 6.50 +/− 2.07) to mild-moderate (VAS 4.24 +/− 2.91) after 1 month and to mild (VAS 1.00 +/− 1.41) after 3 months treatment but was, however, non-significant (*p* > 0.05), likely due to small sample size.

A study based on preliminary data [61] investigated the effect of cannabis consumption on the diverse analgesic outcomes derived from opioids among African American and White cancer pain patients. A tendency of the African American patients to experience pain of higher severity and lower pain relief from opioids compared to White patients was observed. This has been stated to be caused by analgesic undertreatment due to among other reasons low adherence to prescribed analgesics in the African American population. The study indicated that among the included patients (N = 136) 30% consumed cannabis to manage their cancer pains. Cannabis did not exert a significant pain relief but moderated the racial disparities among the African American and White patients in relation to pain relief reduction.

A pilot RCT (N = 30) was recently published, to our knowledge the first in this field, assessing the impact of medicinal cannabis on pain sensation and opioid consumption and the associated safety and required dosing in cancer patients in disease stage IV [52]. The study found that medicinal cannabis was well-tolerated, caused improved pain control and lower opioid consumption in the patients during a 3-month treatment period.

The abovementioned studies, except for the pilot trial by Zylla et al. [52], are not interventional clinical studies following RCT design protocols, but are merely observational based on prospective/retrospective surveys and interviews.

Consequently, they do not provide high-quality evidence but can be perceived as real-world indications of the therapeutic benefits that medicinal cannabis products may offer some cancer pain patients. This can provide loose guidance for potential beneficial directions that future clinical RCTs can consider following.

### 4.2. Clinical Studies of the Cannabis-Based Medication Sativex^®^

The main clinical study characteristics and outcomes are summarized in Table A2.

Sativex^®^ is a standardized cannabis-based medication containing balanced amounts of THC and CBD; 2.7 mg and 2.5 mg, respectively, per spray pump actuation (100 μL) [10].

The first randomized controlled trial (RCT) investigating the analgesic effect of Sativex^®^ in advanced cancer patients (N = 177) being treatment-resistant to opioids was conducted in 2010 [41]. The clinical outcomes were compared against a THC extract (2.7 mg THC per spray/100 μL; carrier liquid not reported) and placebo. Sativex^®^ provided significant (*p* = 0.014) pain relief, with 43% reporting more than 30% reduction in numerical rating scale (NRS) pain score, compared to 21% in the placebo group (*p* = 0.006). The THC extract provided non-significant (*p* = 0.245) pain relief. An extension trial [62] found consumption of Sativex^®^ to be well-tolerated for at least 5 weeks treatment duration. Another RCT conducted by Portenoy et al. [63] evaluated the analgesic effect of Sativex^®^ dose escalations in cancer patients refractory to opioids. Sativex^®^ was administered at different dosage levels, i.e., low (1–4 sprays/day), medium (6–10 sprays/day) or high (11–16 sprays/day) during a 5-week treatment-period. The study found that low to medium dosing of Sativex^®^ exerted improved pain relief compared to placebo. Adverse effects were dose-related, with the Sativex^®^ high dosage group experiencing unfavorable adverse effects, with dizziness and vomiting being prevalent, compared to placebo and low-medium dosage groups. In addition, two RCTs were conducted by Fallon et al. in 2017 [64], which additionally investigated the analgesic effect of adjunctive Sativex^®^ treatment in opioid refractory cancer patients in the U.S. Endpoint percentage improvement in pain score (>30%) was not met but patients did however show improvement in quality of life based on questionnaires. At the baseline of the first trial, subgroup analyses revealed that patients below 65 years of age reported lower opioid consumption and higher pain scores. Sativex^®^ exerted significant (*p* = 0.04) pain relief in this subgroup population. This tendency was hypothesized by the researchers to be caused by the lower background opioid consumption resulting in a lack of opioid receptor downregulation thus allowing a higher synergistic effect among the opioid and cannabinoid receptors to occur, potentially leading to an increased pain relief from Sativex^®^ consumption, which was outlined within the 1st RCT by Fallon et al. [64]. Another RCT [65] was conducted, showing non-significant (*p* = 0.09) pain reduction from adjunctive Sativex^®^ consumption compared to placebo in cancer patients refractory to co-consumed optimized opioid treatment. However, it was stated by the researchers that Sativex^®^ might be a beneficial analgesic in cancer patients consuming low doses of opioids. Again, a tendency for significant beneficial effects of Sativex^®^ within a subgroup of U.S. patients was observed upon secondary endpoints, such as average and worst pain, sleep, and background opioid consumption patterns. It was additionally observed that U.S. patients at baseline consumed lower doses of opioids.

A pilot RCT (N = 16) investigating exclusively the analgesic effects of Sativex^®^ on chemotherapy-induced neuropathic pain was reported by Lynch et al. [66] The study found that Sativex^®^ induced a clinical non-significant relief in pain intensity (stated as a two-point or greater pain reduction on NRS-PI). However, a responder analysis revealed a tendency in favor of Sativex^®^ compared to placebo. It was concluded that Sativex^®^ is worth investigating further in big RCTs in relation to specifically chemotherapy-induced neuropathic pain.

Recently, a systemic review and meta-analysis was conducted [67], covering six of the abovementioned RCTs investigating the analgesic effects of Sativex^®^ on cancer pain. The meta-analysis only included phase II and III trials thereby excluding the study by Lynch et al. [66] due to its focus on specifically CINP and the low number of test persons included. Using pooled RCT data from the five included studies [41,63,64,65] (i.e., Johnson et al., Portenoy et al., Fallon et al. study 1 and 2 and Lichtman et al., respectively) the meta-analysis failed to establish any significant effect of Sativex^®^ on pain reduction, with only the phase II study by Johnson et al. [41] showing benefit in the primary outcome of significant pain reduction from Sativex^®^ consumption. Adverse effects with following dropouts were commonly observed across the studies, mainly dizziness, nausea, vomiting and somnolence. The risk of bias within the included studies of the meta-analysis were assessed by Boland et al. to be low in all domains, based on the Cochrane Collaboration risk of bias tool for RCTs [68]. It was thereby decided that they could not support recommendation of Sativex^®^ as analgesic to cancer patients. However, the meta-analysis’ forest plots assessing the change in pain intensity do show a tendency in favor of Sativex^®^ over placebo, which is supported by the outcomes of the individual RCTs as outlined in Table A2 [67]. Additional systemic reviews and meta-analyses [69,70] performed prior to the one by Boland et al. reached similar conclusions, thereby supporting the outcomes outlined by Boland et al. [67].

Several study limitations were found associated with the included RCTs, such as self-reported pain through VAS, NPS, NRS and NRS-PI scores (which does not detect the complexity related to cancer pain), inaccurate usage of the oromucosal spray (affecting absorption and clinical outcome), high patient withdrawal and mortality rates (affecting the statistical outcome), potential underdosing of cannabis-containing products to avoid potential adverse effects (sub-optimal cannabis analgesia potential obtained) and maintenance of background opioid consumption in several studies (increasing risk of adverse effects) [2,67]. It was stated that due to these methodological limitations, among others, the RCTs were rated as overall low-quality. Tateo et al. based this on a Jaded scale (assessing three components: randomization, blinding and accounting for attribution or dropouts). Thus, it was stated that high-quality evidence is needed to support a stronger conclusion for recommending Sativex^®^ in cancer pain [69].

### 4.3. Administration and Dosing Guidance

Clinical studies have yet to address specific pharmacological factors associated with the administration and dosing of medicinal cannabis. The clinical studies covered by this review do, however, contain data on route of administration, dose, and dosing frequency, as summarized in Table A1 and Table A2. These data can potentially contribute as empirical real-world guidance in the prescription of medicinal cannabis within a clinical setting.

Only general observations regarding administration and dosing parameters were outlined in the clinical studies focusing on medicinal cannabis (i.e., full-spectrum), see Table A1. This means that knowledge regarding safety and efficacy related to these products is based on low-quality evidence. Different cannabis cultivars were preferred among the cancer patients, ranging from high THC, equal THC:CBD and high CBD cultivars, with a general tendency of THC providing optimal analgesic effects according to patients. The study by Nimalan et al. [60], found the initial median consumed daily doses of CBD and THC were reported to 32.0 mg (ranging from 20.0–384.0 mg) and 1.3 mg (ranging from 1.0–16.0 mg), respectively. Zylla et al. [52] found that medicinal cannabis products in general were consumed in daily average doses of 34 mg THC and 17 mg CBD, during a 3-month period. Aviram et al. [59] found that long-term 6-month consumption of especially THC-rich oil extracts resulted in mild to modest statistical improvements on pain, among other symptoms. Monthly THC dosing increased during the 6-month period, while monthly CBD dosing did not significantly increase.

A systematic review was conducted with the aim to elucidate specifically the existing pharmacological evidence of medicinal cannabis administered to cancer patients [19]. Four clinical studies [41,62,63,66] (i.e., Johnson et al. study 1 and 2, Portenoy et al. and Lynch et al., respectively), all of which have been included in this review, focused on Sativex^®^’ analgesic effects on cancer pain. The oromucosal spray Sativex^®^ contains, as previously mentioned; 2.7 mg THC and 2.5 mg CBD per 100 μL, which is equivalent to one actuation of the spray pump [63]. Data from these studies confirm that Sativex^®^ at low (1–4 sprays/day, i.e., 2.7–10.8 mg THC and 2.5–10.0 mg CBD) and medium (6–10 sprays/day, i.e., 16.2–27.0 mg THC and 15.0–25.0 mg CBD) doses are well tolerated with sustained efficacy over a 5-week period [63]. However, consumption of high doses (11–16 sprays/day, i.e., 29.7–43.2 mg THC and 27.5–40.0 mg CBD) resulted in adverse effects, confirming dose-dependency and a general wide therapeutic window [63,71]. In relation to CIPN specifically it was observed that an average administration of 4.5 sprays/day (i.e., 12.15 mg THC and 11.25 mg CBD) exerted analgesic effects, however ranging from 2–20 sprays/day (i.e., 5.4–54.0 mg THC and 5.0–50 mg CBD) [66].

A recently published article [72] outlined existing consensus recommendations regarding dosing and administration of medicinal cannabis prescribed to chronic pain patients, based on the global clinical experience of twenty experts. Chronic pains can be of neuropathic, nociplastic, inflammatory and mixed origins. As several of these pain forms are implicated in the etiology of cancer pain, these empirical data can be considered relevant as guidance for prescription. Despite different protocols, it was generally recommended to start with a CBD-rich cannabis product at a daily dose of 10 mg, which can then be up-titrated until an effect is observed by the patient, to a maximum of 40 mg/day. Thereafter it may be considered to add 2.5 mg THC and up-titrate until a maximum of 40 mg/day. A more rapid approach is to start with an equal ratio THC:CBD product at 2.5–5 mg/day of each cannabinoid until a maximum of 40 mg/day THC is reached. The recommendation to “start low and go slow” should thus be followed, with gradual increases of the dose until optimal therapeutic effect is obtained with a minimum of side- and adverse effects. It has been proposed that consumption of high doses of THC (i.e., approx. 30–40 mg/day) require slow titration over a period of approximately two weeks to induce tolerance in the patient to especially psychoactive adverse effects [72,73]. It is however important to keep in mind that the therapeutic outcome is individual and highly depends on the patients’ individual tolerance and underlying endocannabinoid tone, the route of administration and dosing patterns [73,74].

### 4.4. Safety Profile and Opioid-Sparing Effects of Cannabinoids

Cannabis in general possess a superior safety profile compared to, e.g., opioids, with no reported death due to overdosing of cannabis, expected to be due to sparse CB1R localization within the brainstem region containing the respiratory centers [73,75]. Side and adverse effects related to consumption of cannabis products are mainly THC-mediated, dose-dependent and transient and mostly mild to moderate in severity, as outlined in Table A1 and Table A2. The general recommendation related to administration and dose titration “start low and go slow” aims to minimize these effects, with co-consumption of THC and CBD further aiding in preventing this. However, patients have been observed to develop tolerance to these potentially occurring adverse effects, e.g., psychoactive, cognitive, dizziness, nausea, and drowsiness symptoms, over a period of only a few days without developing tolerance towards the therapeutical effects. In contrast to opioids, this allows most patients to remain on an almost stable dose for years [73]. It has been proposed that the higher tendency of adverse effects causing withdrawal within some of the short-term studies compared to longer-term observational studies might be caused by a too rapid titration within naïve cannabis consumers [2], which may negatively impact the clinical outcomes. Withdrawals reported in some of the studies could be due to many of the patients being in an advanced cancer disease state (some studies reporting up to 25% mortality rate). Cancer disease is often associated with comorbidities and several disease-related symptoms, with potential sub-optimal dosing of, e.g., cannabinoids or opioids thereby not properly alleviating the patients’ symptoms. However, analgesic effects of THC on particularly neuropathic pains in general have been observed to occur at much lower plasma levels than the levels associated with adverse effects in the form of particularly euphoria-associated adverse effects within clinical settings [72].

The Sativex^®^ RCTs addressed in this review (Table A2) have all investigated the analgesic effect as adjunctive to opioid treatment in optimized opioid-resistant cancer patients. No clinical studies have yet examined the direct comparable analgesic effect of Sativex^®^, or medicinal cannabis, versus opioids. Several of the studies kept the patients on stable opioid dosing, while others allowed for dosing regulations during the study period. Additive and synergistic analgesic effects of cannabinoid- and opioid co-consumption have been observed, with concurrent decrease in adverse effects and a lower occurrence of developing addiction towards opioids [73]. This was for example illustrated within the RCT performed by Johnson et al. [62], where long-term usage of Sativex^®^ in cancer patients refractory to opioids did not lead to an increase in dose of either Sativex^®^ or opioids over months, despite disease progression. The opioid-sparing potential of cannabinoids was recently assessed within a systemic review and meta-analysis by Nielsen et al. [35] Based upon four RCTs [41,64,65] (i.e., Johnson et al., Fallon et al. study 1 and 2 and Lichtman et al., respectively) including cancer pain patients, which all have been included within this review, Sativex^®^ consumption was not observed to significantly (*p* = 0.30) affect opioid background dose co-consumption when study data were pooled in the meta-analysis. These findings were supported by another systemic review and meta-analysis performed by Noori et al. [76] based on the same RCTs, where the same tendencies were observed.

Based on the clinical observation-based studies of medicinal cannabis included in this review (Table A1), a tendency for many cancer patients being able to reduce or terminate opioid co-consumption is evident. The pilot RCT by Zylla et al. [52] additionally indicated a potential opioid-sparing effect from medicinal cannabis consumption, where the group receiving cannabis delayed (standard oncology treatment first 3 months, then initiation of cannabis treatment at 4th month for 3 months) increased opioid consumption, compared to the group receiving cannabis early (from the initiation of study for 3 months). Pawasarat et al. [56] found a reduction of 33% in daily opioid background consumption in patients co-administered with medicinal cannabis. In support of this, a U.S. epidemiological study found that states with access to medicinal cannabis also recorded a decrease in opioid overdose-related mortality [77]. A recently published survey-based study by Pritchett et al. [78] support these findings as it found that medicinal cannabis consumption led to reduction or cessation of opioid usage, which was confirmed by 79% of the participating patients (N = 2183), including cancer patients suffering from pain and mental health ailments (n = 85). Several patients even stated to prefer medicinal cannabis as it allowed for a better functioning in their daily life.

These potentially synergistic effects of opioid-cannabinoid co-consumption are supported by pre-clinical studies and knowledge of similarities among cannabinoid and opioid receptors, their localizations within the body and the intracellular mechanisms they induce (see Section 2., i.e., *The endocannabinoid system and its implication in cancer pain* of this review). In summary, these studies suggest potential beneficial effects of co-administering cannabinoids as adjunctive analgesic in cancer patients not satisfactorily pain relieved from their opioid therapy.

## 5. Challenges and Barriers in Medicinal Cannabis Research

Performing research on the therapeutic effects of medicinal cannabis is highly complex and associated with several challenging factors such as; administration form impacting bioavailability, heterogenous compositions and potencies of the products, potential of synergistic interactions between the multiple bioactive compounds, heterogenous pain etiology, patient genetics impacting the ECS functionality and hepatic metabolism of cannabinoids determining the individual patients’ tolerance against cannabis, etc. These factors can all impact both the pharmacokinetic and dynamic effects occurring within the body, thereby introducing noise in the evaluation of clinical response ultimately leading to poor statistical power of such studies. Collectively this complicates the performance and interpretation of clinical data. The fact that high-fat dietary products can lead to an increased absorption of the cannabinoids, due to their lipophilic chemical nature, also impacts the resulting therapeutic effects. This underpins the relevance of a personalized approach for prescribing medicinal cannabis [19].

### 5.1. Regulatory Barriers

Regulatory barriers restrain cannabis research, especially due to its classification as a Schedule I substance drug in the U.S., i.e., a narcotic. This has limited researchers’ access to medicinal cannabis products and instead shifting much of the research to single cannabinoids leaving a gap between the two research areas [13]. Focusing on a single cannabinoid also relates better to the *“one drug one target”* approach associated with the classical pharmaceutical research field, also carrying the inherent benefit of a standardized product with less statistical noise introduced to the clinical study.

### 5.2. Several Methodological Challenges

Several methodological challenges also pertain to the research field of medicinal cannabis. Drug delivery technology, route of administration and dosing regimen impact bioavailability and ultimately clinical outcome. The mechanism of actions exerted by the bioactive compounds available within the body differ among individuals due to individual tolerances against the compounds and underlying individual tones of endocannabinoids [73]. THC is associated with adverse effects, if consumed in high doses, due to its psychoactive effects in the body, which the individual can build tolerance against over time [13,73,79]. These factors, among others, collectively affect and determine the resulting effect and clinical outcome, ultimately making it challenging to compare across studies.

#### 5.2.1. Standardization of Materials

Standardization of materials within clinical cannabis research is not completely possible due to cannabis being a biological material product with different cannabis cultivars possessing unique compositions of biologically active molecules. In addition to the genetics, cultivation factors and extraction technology also influence the composition of the final product [79]. The heterogeneity of cannabis cultivars used as raw material for production of different medicinal cannabis products have made it challenging for the research to conduct consistent and reproducible clinical pharmacological studies, and it is therefore highly recommended to fix the specific cultivar throughout the course of the entire study. One should also exercise caution when comparing data (e.g., through pooling of data or extrapolation) between products manufactured from different cultivar material as failure to do so can lead to erroneous conclusions [79,80].

#### 5.2.2. Placebo-Control

Placebo-control is part of the gold-standard methodology associated with RCTs, which aids in blinding the study participants to minimize the risk of bias. As the risk of bias is generally high in the context of clinical cannabis research, a need for cautious interpretations of clinical outcomes is necessary [81]. However, it has been difficult to develop a true placebo of medicinal cannabis that study participants are unable to disclose. This is due to the psychoactive effects and characteristic aroma associated with cannabis consumption, especially in relation to THC-rich cultivars [80]. Some placebo products have been developed through removal of the primary cannabinoids such as THC and CBD from the formulation, although these have often been found to retain residual cannabinoids and terpenoids, among others. As these compounds may also be biologically active, their status as neutral ingredients suitable for placebo production can be questioned [13].

#### 5.2.3. Clinical Data Evaluation

Clinical data evaluation can be challenged by several factors such as the lack of fully standardized products containing varying concentrations of bioactive compounds and thereby inconsistencies in administration and dosing. Observational studies, relying primarily on self-reporting of clinical outcomes, carries a substantial risk of data being inaccurately reported. Additionally, missing information of potential poly-pharmaceutical consumption can cause confounding effects making it difficult to determine the therapeutic effect specifically attributed to the treatment with medicinal cannabis [13].

This review has focused solely on medicinal cannabis (i.e., full-spectrum products) and the natural cannabis extract product Sativex^®^. Sativex^®^ has been more extensively researched in RCTs for its analgesic effects on cancer pain. This may probably be due to its status as a broad-spectrum product, meaning that it has been purified beyond just the dewaxed extract providing a better standardization than a full-spectrum product. Sativex^®^ has furthermore received regulatory approval, indicating that the product has demonstrated consistency in the chemical profile of the drug substance (i.e., biomass) and drug product (i.e., Sativex^®^) within and across batches [82]. Medicinal cannabis on the contrary is a challenging material to include in RCTs, as the clinical effects originate from non-specific targeting at a myriad of localizations within the body by the interaction of multiple compounds and receptor responses. This is somewhat of a paradox considering that the lack of standardization provides the foundation for the proclaimed entourage effect.

## 6. Conclusions

Based on published research literature included in this selective review, it is evident that there is a clear lack of high-quality interventional studies of gold-standard RCT design investigating the usage of medicinal cannabis (i.e., full-spectrum) as analgesic in cancer patients. However, some observation-based studies have been performed, which in general indicate analgesic potentials for most cancer patients. Higher quality clinical research, i.e., RCTs have been performed regarding the cannabis-based medicinal product Sativex^®^. The RCTs—as stand-alone studies—confirm some analgesic effect with a good safety profile. However, a recent systematic review and meta-analysis based upon these RCTs failed to demonstrate a significant analgesic effect of Sativex^®^ in cancer patients.

Clinical studies performed by gold standard methods are in general scarce and needed, as these—depending on clinical outcome—will allow for the conduction of solid evidence-based dosing and administration guidelines of medicinal cannabis products associated with the optimal efficacy and safety profiles for chronic pain cancer patients [83]. However, the studies outlined in this review show indications of dose and administration regimens beneficial in providing pain relief for some cancer patients. As such, consensus recommendations exist [72] in relation to chronic pain in general, based on international experts’ clinical experiences with the prescription of medicinal cannabis and cannabis-based products (see Section 4.3., i.e., *Administration and dosing guidance* of this review).

Clinical evidence is currently not strong enough to determine if medicinal cannabis, or cannabinoid-based products, provide adequate, equal, or increased analgesic effects compared to available conventional treatments such as opioids across the population of cancer patients. The opioid-sparing effects that these products have been observed and proposed to exert in some patients could offer a potential advantage to many patients experiencing opioid treatment resistance and adverse effects [1]. However, the research field comes with a line of barriers and faces several challenges, as discussed in this review. These need to be tackled to improve *directions for use* of medicinal cannabis products and ultimately provide the best possible treatment plan for the highly heterogenous cancer patient population, in line with a personalized treatment approach.

The cannabis plant has been referred to as a *treasure trove*, as it contains multiple bioactive compounds possessing therapeutic potentials [84]. This holds the potential of developing medicinal products based on compounds possessing targeted multiple therapeutical effects, thereby affecting several symptoms within the individual patient all at once. This potentially offers a comprehensive treatment option for, e.g., cancer patients often suffering from several symptoms and a complex underlying disease pathophysiology, thereby avoiding polypharmacy related adverse effects impacting the quality of life of patients ([2], pp. 143–145, [43]). Research exploring the therapeutic potential of cannabinoids currently moves in different directions: medicinal cannabis (i.e., full-spectrum) and cannabinoid-based medicinal products, based on one or several cannabinoids derived from either the cannabis plant or synthetically manufactured [79]. Independent of the product type, they must follow established standards regarding quality, safety and efficacy, to be able to develop into authorized medicinal products. This offers exciting opportunities for potentially a whole new portfolio of medicinal products to be developed in the future; to the benefit of not only cancer pain patients but potentially all disease populations [82].

## Data Availability

Not applicable.

## Appendix A

**Table A1:** Clinical data summary of medicinal cannabis products (i.e., full-spectrum).

**Schleider et al., 2018 [53]** Prospective analysis of safety and efficacy of medical cannabis in large unselected population of patients with cancer	
**Location**	Israel
**Aim of study**	Efficacy and safety of different medicinal cannabis cultivars in cancer patients, suffering from pain among other symptoms Primary outcome: Treatment success at 6-month follow-up
**Study design**	Prospective, single-center (N = 2970 initially, N = 1211 after six months) Patient population: Cancer patients treated with medicinal cannabis in 2015–2017 within a cannabis clinic (Tikun Olam), 51.2% had stage IV cancer, of which 77.7% were in pain with median intensity 8–10 on a visual analogue scale (VAS, 0–10) Numeric Rating Scale used to measure pain (NRS, 0–10, 0 = no pain and 10 = worst pain imaginable) 1- and 6-month follow-up, additional 1- and 2-year follow-up based on patient status
**Drug, administration, and dosing**	Medicinal cannabis 16 dif. cultivars w. 4 main types: 1: THC-rich indica (22–28% THC and <0.5% CBD), consumed by 91.8% 2: THC-rich sativa (No CBD), consumed by 60.5% 3: Equal THC:CBD (15%), consumed by 23.2% 4: CBD-rich (20% CBD and <1% THC), consumed by 32.4% Oil and/or inflorescence (i.e., flower, capsule and cigarette), 44% had license for both types
**Primary outcomes in pain**	60.6% (N = 1211) responded at 6-month follow-up, where 95.9% reported improvement, 3.7% no change and 0.3% deterioration of disease condition 52.9% reported high pain (VAS score 8–10) at baseline, with 4.6% reporting this after 6-month treatment with medicinal cannabis (*p* < 0.001)
**Side effects/** **adverse effects**	Most common side effects reported by n = 362 at 6-month follow-up: Dizziness, dry mouth, increased appetite, sleepiness and psychoactive effects, but were minor and easy to cope with Some patients had terminated treatment at 6-month follow-up due to side effects
**Additional notes**	Most patients consumed more than one cannabis cultivar, with 91.8% using THC-rich indica At 6-month follow-up, 35.1% had reduced their consumption of background medicinal products, of which 33.9% consumed opioids and 36.0% reported termination of intake 18.7% reported good quality of life at baseline, with 69.5% reporting this after 6 months (*p* < 0.011) At 6-month follow-up, 24.9% (n = 902) had died, and 18.8% (n = 682) terminated their treatment Reason for termination of treatment at 6-month follow-up (n = 249): No need for cannabis treatment, no therapeutic effect and side effects, but 52.2% reported moderate improvement in symptoms Medical cannabis in the palliative care of cancer patients well-tolerated, effective and safe treatment option to help cope with cancer symptoms
**Aviram et al., 2020 [55]** Short-term medical cannabis treatment regimens produced beneficial effects among palliative cancer patients	
**Location**	Israel
**Aim of study**	Assess and compare short-term efficacy and safety of different medicinal cannabis cultivar products in cancer patients, including pain patients
**Study design**	Prospective (N = 108), short-term, follow-up after 1 month
**Drug, administration, and dosing**	Medicinal cannabis 3 different cultivar groups: I: THC-rich, consumed by 52% (n = 56) II: Equal THC:CBD, consumed by 30% (n = 33) III: CBD-rich, consumed by 18% (n = 19) Sublingual oil OR inflorescence inhalation, with sublingual oil most common for CBD and THC:CBD cultivars Approx. 20 g medical cannabis consumed in 1 month, with significant different doses of THC and CBD: Type I consumed dose per month: 600 (400–725) mg CBD and 3000 (2000–3600) mg THC Type II consumed dose per month: 2000 (1500–2000) mg CBD and 2000 (1400–2000) mg THC Type III consumed dose per month: 2000 (2000–3000) mg CBD and 1000 (600–1000) mg THC
**Primary outcomes in pain**	Significant (*p* < 0.05) improvement in intensity of affective and sensory pain. No difference among the different cultivars on pain intensity. CBD-rich cultivars should be considered and recommended compared to THC:CBD or THC-dominant, as it is shows better treatment in cancer patients with high physical symptom burden.
**Side effects/** **adverse effects**	Most adverse effects associated with type I, followed by Type II and then type III, indicating adverse effects are related to cultivars rich in THC n = 14 terminated treatment due to adverse effects (dizziness, hallucination, anxiety, faint, fatigue, nausea, drowsiness, restlessness)
**Additional notes**	No difference among the different cultivars on secondary outcomes such as sleep and psychological symptoms CBD-rich oil induced similar effect as THC-dominant cultivars in secondary outcomes THC-rich oil shows significant (*p* < 0.05) superiority compared to CBD-rich in relation to beneficial effects on sleep duration Improvement in quality-of-life sensation was experienced by several patients Analgesic background co-consumption decreased, although not significant across groups No significant impact on the safety profile of all cultivars Overall mild improvement in symptoms regardless of cultivar
**Pawasarat et al., 2020 [56]** The Efficacy of Medical Marijuana in the Treatment of Cancer-Related Pain	
**Location**	New Jersey, U.S.
**Aim of study**	Characterization of medical marijuana (i.e., medicinal cannabis) role in cancer symptom relief and on opioid consumption
**Study design**	Retrospective (N = 232; n = 137 medicinal cannabis consumers and n = 95 non-medicinal cannabis consumers, both groups on background opioids)
**Drug, administration, and dosing**	Medicinal marijuana (i.e., medicinal cannabis)
**Primary outcomes in pain**	Medicinal cannabis consumption improved symptoms, including pain, despite reduction in opioid intake, while non-medicinal cannabis consumers increased opioid intake
**Side effects/** **adverse effects**	Opioid-sparing effect of medicinal marijuana cause subsequent less adverse side effects by opioids
**Additional notes**	Medicinal cannabis as adjunctive to opioids provided analogue symptom relief without burden of opioid dose escalation Medicinal cannabis should be considered adjuvant treatment in palliative care
**Zarrabi et al., 2020 [57]** Perception of Benefits and Harms of Medical Cannabis among Seriously Ill Patients in an Outpatient Palliative Care Practice	
**Location**	Georgia, U.S.
**Aim of study**	Seriously ill patients’ perceptions of benefits and harms related to medicinal cannabis within the palliative care
**Study design**	Online survey N = 101 (76% cancer patients)
**Drug, administration, and dosing**	Medicinal cannabis Patients could choose more than one product Types of consumed products: Low-dose THC (<5%) oil (49.5%) Mixed THC/CBD oil (33.7%) Non-oil (20%) Pure CBD oil (14.9%) High-dose THC (>5%) oil (12.9%) THCA oil (2%) Unknown content of oil (8.9%) Administration forms: Oils most frequently administered orally (61%) or by vaporizing (49%) Smoking (23%) Edibles (16%) Topical (2%)
**Primary outcomes in pain**	96% perceived considered cannabis important to their pain management Seriously ill patients consume cannabis for curative intention and pain control as well as other symptoms
**Side effects/** **adverse effects**	Side effects were minimal, with drowsiness (27.6%) being most common
**Additional notes**	Cannabis consumption improved other symptoms such as anxiety, depression relief, increase energy, nausea and well-being 62.2% considered cannabis important to their cancer curative treatment 65% co-consumed opioids
**Zylla et al., 2021 [52]** A randomized trial of medical cannabis in patients with stage IV cancers to assess feasibility, dose requirements, impact on pain and opioid use, safety, and overall patient satisfaction	
**Location**	Minnesota, U.S.
**Aim of study**	Safety, efficacy, and dosing related to medicinal cannabis usage in cancer patients
**Study design**	Pilot RCT (N = 30: n = 15 early start cannabis and n = 15 delayed start cannabis, whereof n = 21 initiated cannabis treatment during the trial) Early cannabis group consumed cannabis for 3 months Delayed cannabis group received oncology standard care without cannabis for first 3 months, then offered cannabis for 3 months Pain measured on VAS (0–10) Blood samples at 3-month
**Drug, administration, and dosing**	Medicinal cannabis Patients advised to initiate dose of 2.5–5 mg THC and CBD, titrating to max. 30–40 mg THC and CBD per day for 2–4 weeks Mean daily allotment of THC (34 mg) and CBD (17 mg) at 3-month Oral administration most common (26% solid dosage and 25% liquid), inhaled (35%) and topical (14%) Main product types prescribed: High THC:CBD ratio (63%), balanced THC:CBD ratio (30%) and high CBD:THC ratio (7%)
**Primary outcomes in pain**	More patients in early cannabis group experienced opioid reduction and improved personalized pain goal
**Side effects/** **adverse effects**	No adverse effects reported and no serious safety problems
**Additional notes**	Interest in study was high Early cannabis group stable opioid consumption and delayed cannabis group increased average daily opioid consumption Blood samples revealed mean plasma levels of THC and CBD to be 7.3 ng/mL and 6.0 ng/mL, respectively Patients experienced high satisfaction and well-tolerated response
**Meghani et al., 2021 [61]** Impact of Cannabis Use on Least Pain Scores Among African American and White Patients with Cancer Pain: A Moderation Analysis	
**Location**	Pennsylvania, U.S.
**Aim of study**	Cannabis use for medicinal pain purposes impact on opioid intake and pain score in African American and White patients
**Study design**	Preliminary data from ongoing longitudinal study N = 136 (n = 49 African American and n = 87 White), with 30.1% reporting cannabis use for cancer pain Patients reported outcomes at baseline, 1-, 2-, 3- and 5 months Pain scored on scale 0–10 (0 = no pain and 10 = pain as bad as you can imagine)
**Drug, administration, and dosing**	Medicinal cannabis
**Primary outcomes in pain**	Cannabis use did not significantly impact pain, but acted as a significant moderator as it was able to moderate the relationship among race and pain score (*p* = 0.03) Absence of cannabis use was associated with higher pain scores in African Americans compared to Whites, but these racial disparities were not observed when cannabis was consumed
**Side effects/** **adverse effects**	Few patients experienced medication side effects
**Additional notes**	Disparities in prescription of analgesics such as opioids for cancer pain among African Americans and Whites Role for cannabis in pain management and reduction of racial disparities
**Schleider et al., 2022 [54]** Adherence, Safety, and Effectiveness of Medical Cannabis and Epidemiological Characteristics of the Patient Population: A Prospective Study	
**Location**	Israel
**Aim of study**	Characterize medicinal cannabis patient population and identify treatment adherence, safety and effectiveness
**Study design**	Prospective (N = 10.000 (approx.), with n = 3.173 cancer pain patients) Patient population: Patients prescribed medicinal cannabis, with cancer patients most frequently prescribed it Study period: 6 months Based on database data included in Schleider et al., 2018 study, with overlap of patients
**Drug, administration, and dosing**	Medicinal cannabis diff. chemovars: Erez: Indica (18% THC, 0% CBD) Alaska: Sativa (18% THC, 0% CBD) Avidekel: Indica (15% CBD, 0.5% THC) Smoke, vaping and sublingual oil administrations Average dosing: 2.4–4.3 administrations per day Average cannabinoid dose per administration: 5.2–54 mg THC and 6.7–39 mg CBD
**Primary outcomes in pain**	Non-specific pain was the second most common reason for medicinal cannabis prescription (29.3%) Out of N = 4166 pain assessed patients; 62.0% report high pain intensity at baseline (VAS score 8–10), with only 5.0% reporting this after 6 months treatment (*p* < 0.001) In patients assessed for pain, 7.3% reported deterioration in pain score, 17.8% reported no change in pain score and 74.7% reported improvement in pain score (64.3% showing more than 30% reduction in pain intensity and 47.2% more than 50% reduction in pain intensity)
**Side effects/** **adverse effects**	Low incidences of serious adverse effects Common side effects reported by 34.2%: Dizziness, dry mouth, increased appetite, sleepiness, and psychoactive effects
**Additional notes**	70.6% experienced treatment success after 6 months Medicinal cannabis treatment associated with high adherence and decrease in pain Improvement in quality of life, reported by n = 4143, with 12.9% stating good at baseline and 69.9% stating this after 6 months treatment with cannabis (*p* < 0.001) Effects on opioid co-consumption: 45.5% remained on same dose, 38.8% terminated and 13.7% decreased opioid consumption after cannabis treatment initiation
**Aviram et al., 2022 [59]** The Effectiveness and Safety of Medical Cannabis for Treating Cancer Related Symptoms in Oncology Patients	
**Location**	Israel
**Aim of study**	Assess efficacy and safety of medicinal cannabis for treating cancer-related symptoms, including pain
**Study design**	Prospective, longitudinal, large-scale cohort Long-term: 1, 3 and 6-month follow-ups N = 324 (n = 212, n = 158 and n = 126 during follow-up after 1, 3 and 6 months, respectively)
**Drug, administration, and dosing**	Medicinal cannabis Median monthly dose 20 g: THC monthly doses increased from median 2000 mg (1000–3500) to 3000 mg (2000–4000) CBD monthly doses did not change significant Oil extract most common administration form (41%) consumed sublingually THC-rich cultivar consumed most frequent
**Primary outcomes in pain**	Significant changes from baseline till after 6-month treatment in, e.g., average weekly pain intensity reduced by median 20% from 7(3–9) to 5 (3–7) (*p* < 0.001) 36% reported more than 30% pain intensity reduction from baseline till 6-month follow-up 20% reported no change or increase in pain intensity Least pain and worst pain intensity as well as sensory pain and affective pain were also reduced significantly (*p* < 0.001)
**Side effects/** **adverse effects**	No serious adverse effects, but adverse effects were common
**Additional notes**	Long-term medicinal cannabis consumption induces mild to modest statistical improvements in pain Reduction in opioid and other conventional analgesics intake Quality of life improved significant from baseline till 6-month follow-up by median 14% Medicinal cannabis generally perceived as safe and can reduce symptom burden, including pain
**Nimalan et al., 2022 [60]** UK Medical Cannabis Registry palliative care patients cohort: initial experience and outcomes	
**Location**	United Kingdom (U.K.)
**Aim of study**	Summarize initial experiences and outcomes of a UK patient subgroup prescribed medicinal cannabis as part of palliative care, cancer pain and chemotherapy-induced nausea and vomiting, including effects on quality of life and clinical outcome
**Study design**	Prospective, preliminary cohort N = 16 (n = 12 palliative care, n = 3 cancer-pain and n = 1 chemotherapy-induced nausea and vomiting) Pain assessed by VAS scale 1-month and 3-month follow-up
**Drug, administration, and dosing**	Cannabis-based medicinal product, including the broad-spectrum 50 mg/mL CBD oil (Adven 50, Curaleaf International) and 20 mg/mL THC oil (Adven 20, Curaleaf International) Most patients (87.5%) were prescribed two different oil-based cannabis-based medicinal products Median daily dose CBD: 32.0 mg (20.0–384.0 mg) Median initial daily dose THC: 1.3 mg (1.0–16.0 mg)
**Primary outcomes in pain**	Improvement in patient reported health outcomes, including pain Pain reduced from severe (6.50 +/− 2.07) to moderate-mild (4.24 +/− 2.91) after 1 month and mild (1.00 +/− 1.41) after 3 months, based on VAS score, but were non-significant (*p* > 0.05)
**Side effects/** **adverse effects**	Few (n = 3) adverse effects being mild to moderate in severity and spontaneous resolved, overall well-tolerated
**Additional notes**	No significant improvements observed due to small sample size
**Sura et al., 2022 [58]** Experience With Medical Marijuana for Cancer Patients in the Palliative Setting	
**Location**	New York, U.S.
**Aim of study**	Review characteristics related to patients receiving medicinal cannabis in palliative care program, while determining barriers to access its usage
**Study design**	Retrospective, single-center (N = 184, of which 51.5% received at least one prescription for medicinal cannabis)
**Drug, administration, and dosing**	Medical marijuana (i.e., medicinal cannabis) Different administration forms such as capsule, vaporizer and solution most common used
**Primary outcomes in pain**	48.14% of medicinal cannabis consumers experienced improvements in pain, 44.95% reduced opioid intake
**Side effects/** **adverse effects**	Adverse effect incidences low (3.72%), with dizziness light-headed and funny feeling reported, one severe adverse effect with dizziness causing collapse Medicinal cannabis in general associated with few adverse effects
**Additional notes**	Medicinal cannabis plays an important role in the palliative care in advanced cancer patients Many barriers remain for effective use of medicinal cannabis 50% consume cannabis for anti-cancer properties

## Appendix B

**Table A2:** Clinical data summary of Sativex^®^ (a cannabis-based medicinal product).

**Johnson et al., 2010 [41]** Multicenter, double-blind, randomized, placebo-controlled, parallel group study of the efficacy, safety, and tolerability of THC:CBD extract and THC extract in patients with intractable cancer-related pain	
**Location**	United Kingdom (U.K.) and Belgium
**Aim of study**	Evaluate efficacy of Sativex^®^ and a THC extract, compared to placebo, on pain inadequate alleviated by opioids in advanced cancer patients
**Study design**	Randomized, placebo-controlled trial (RCT), phase II Double-blind, parallel-group, multicenter N = 177 (patients randomized to Sativex^®^ (n = 60), THC extract (n = 58) or placebo (n = 59) Patient population: Advanced cancer patients with at least moderate severe pain (NRS score 4–10) with background opioid consumption Study period: 2-day baseline, then 2-week treatment Patients self-titrated until optimal dose during week 1 (with max. 50% dose increase per day), then continued this dose until end of study Numerical Rating Scale (NRS) score (0 = no pain and 10 = very bad pain)
**Drug, administration, and dosing**	Sativex^®^ (Nabiximol) oromucosal spray Sativex^®^ content: THC 27 mg/mL CBD 25 mg/mL 1 spray = 100 μL = 2.7 mg THC and 2.5 mg CBD Min. dose: 2.7 mg THC and 2.5 mg CBD Max. dose: 129.6 mg THC 120 mg CBD THC extract content: 2.7 mg THC (1 spray = 100 μL) Max. allowed dosing for both treatments: 8 sprays/3 h OR 48 sprays/24 h Average dosing: 11, 9 and 8 sprays/day in placebo, Sativex^®^ and THC group, respectively
**Primary outcomes in pain**	Statistically significant (*p* = 0.014) pain reduction from baseline in mean pain NRS score from Sativex^®^ compared to placebo
**Side effects/** **adverse effects**	Withdrawal due to adverse effects: Sativex^®^ 17%, THC extract 12% and placebo 3%; Most were of mild-moderate severity and no raised concerns regarding safety of treatment Sativex^®^ and THC extract well-tolerated
**Additional notes**	Double number of patients (n = 23, 43%) in Sativex^®^ group experienced more than 30% pain reduction in NRS score from baseline compared to placebo (n = 12, 21%) THC extract non-significant (*p* = 0.245) with comparable pain reduction effect as placebo (n = 12, 23% vs. n = 12, 21%, respectively) No change across treatment groups from baseline in median dose of background opioid consumption or mean dose of breakthrough medication Sativex^®^ effective as adjunctive analgesic in cancer patients inadequately pain-relieved by opioids
**Portenoy et al., 2012 [63]** Nabiximols for opioid-treated cancer patients with poorly controlled chronic pain: A randomized, placebo-controlled, graded-doses trial	
**Location**	North America, Europe, Latin America and South Africa
**Aim of study**	Evaluate efficacy and safety related to Sativex^®^ consumption in three different doses on pain in advanced cancer patients refractory to opioids
**Study design**	RCT, phase II Double-blind, parallel group, multicenter, graded dose study N = 360 (n = 263 completed study) Patients randomized to low-dose (n = 71), medium-dose (n = 67), high-dose (n = 59) or placebo (n = 66) Patient population: Advanced cancer patients refractory to opioids with background opioid consumption Study period: 5–14 days baseline, then 1-week blinded dose titration, followed by 4-week stable dose treatment Post-study follow-up after 2 weeks
**Drug, administration, and dosing**	Sativex^®^ (Nabiximol) oromucosal spray 3 groups up-titrated blindly to either: Group 1: Low dosing (1–4 sprays/day) Group 2: Medium dosing (6–10 sprays/day) Group 3: High dosing (11–16 sprays/day) Min. dose: 2.7 mg THC and 2.5 mg CBD Max. dose: 43.2 mg THC and 40 mg CBD
**Primary outcomes in pain**	Pain reduction by 30% from baseline on NRS non-significant (*p* = 0.59) for Sativex^®^ vs. placebo Sativex^®^ in low- and medium-dose is an effective and safe analgesic, and thus may be beneficial as adjunctive analgesic in cancer patients refractory to opioids
**Side effects/** **adverse effects**	Withdrawal due to adverse effects: Adverse effects were dose-related, only high-dose Sativex^®^ group unfavorable compared to placebo, while rates of low- and medium-dose groups comparable to placebo
**Additional notes**	Average daily pain reduction from baseline was greater in Sativex^®^ treated groups vs. placebo (*p* = 0.035), specifically in low-dose (*p* = 0.008) and medium-dose (*p* = 0.039) groups Pain score changes occurred in absence of change in background opioid co-consumption Tendency of underdosing in Sativex^®^ groups, which increased with dose increasement, thus most prevalent in high-dose group
**Johnson et al., 2013 [62]** An open-label extension study to investigate the long-term safety and tolerability of THC/CBD oromucosal spray and oromucosal THC spray in patients with terminal cancer-related pain refractory to strong opioid analgesics	
**Location**	U.K. and Belgium
**Aim of study**	Follow-up study of Johnson et al., 2010 Assessing long-term safety and tolerability of Sativex^®^ and a THC extract in terminal cancer pain patients refractory to opioids
**Study design**	Open label, follow-up study, multicenter N = 43 (n = 4 THC and n = 39 Sativex^®^) Patient population: Advanced cancer pain patients refractory to strong opioids, previously enrolled in the RCT trial of Johnson et al., 2010 Study period max. approx. 2 years (657 days) Patients self-titrated over 7–10 days, until symptom relief or max. dose, with max. 50% increase in dose per day Brief Pain Inventory-Short Form (BPI-SF) score (0–10), with decrease indicating improvement, i.e., decrease in pain
**Drug, administration, and dosing**	Sativex^®^ (Nabiximol) oromucosal spray OR THC oromucosal spray, content: 1 spray = 2.7 mg THC Max. allowed dosing: 8 sprays/3 h OR 48 sprays/24 h Min. dose: 2.7 mg THC and 2.5 mg CBD Max. dose: 129.6 mg THC and 120 mg CBD Average dosing Sativex^®^: 5.4 +/− 3.28 sprays/day Average dosing THC extract: 14.5 +/− 16.84 sprays/day
**Primary outcomes in pain**	BPI-SF scores for pain severity and worst pain decreased (i.e., analgesic improvement) with Sativex^®^ Some patients experience continuous analgesic effect (up to at least 5 weeks) from Sativex^®^, without increasing dose hereof or of other co-consumed analgesics
**Side effects/** **adverse effects**	Adverse effects such as dizziness, nausea, vomiting, somnolence, and confusion, with n = 23 and n = 1 withdrawals in Sativex^®^ vs. THC group Long-term consumption of Sativex^®^ well-tolerated
**Additional notes**	Sativex^®^ as adjuvant analgesic beneficial for some cancer patients Many patients co-consumed other analgesics, which were allowed to change during study period
**Lynch et al., 2014 [66]** A double-blind, placebo-controlled, crossover pilot trial with extension using an oral mucosal cannabinoid extract for treatment of chemotherapy-induced neuropathic pain	
**Location**	Canada
**Aim of study**	Investigate efficacy of Sativex^®^ on chemotherapy-induced neuropathic pain (CINP)
**Study design**	RCT, pilot study Double-blind, cross-over N = 18 (n = 9 Sativex^®^ and n = 9 placebo), of which n = 16 completed the study Treatment periods: 1st treatment (Sativex^®^ or placebo). Titration phase (increase by 1–2 sprays/day until max. 12 sprays/day), then 4-week stable dose treatment, followed by down titration (1–2 sprays/day) and a 2-week washout 2nd treatment (Sativex^®^ or placebo) initiation, following same principals as 1st treatment Extension study phase: N = 10 treated with Sativex^®^ up to 6 months Numeric Rating Scale for Pain Intensity (NRS-PI) score, ranging 0–10 (0 = no pain and 10 = very bad pain) Patient population: Neuropathic pain persisting for at least 3 months in cancer patients due to chemotherapy, with average 4–10 score in NRS-PI
**Drug, administration, and dosing**	Sativex^®^ (Nabiximol) oromucosal spray Min. dosing: 1 spray/day Max. dosing: 12 sprays/day Mean dosing: Sativex^®^ 8 sprays/day (ranging 3–12 sprays/day) vs. placebo 11 sprays/day Average dosing in extension phase: 4.5 sprays/day (ranging 2–10)
**Primary outcomes in pain**	No statistic significant differences among Sativex^®^ and placebo group in relation to pain intensity measured on NRS-PI Extension phase of study caused pain reduction from 6.9 to 4.2 from baseline till after 6-month treatment with Sativex^®^
**Side effects/** **adverse effects**	Adverse effects reported by most patients, but majority were mild and transient, none caused withdrawal and very few transient adverse effects reported in the extension phase Most common adverse effects: Fatigue, dizziness, nausea and dry mouth
**Additional notes**	Average 2.6 decrease in NRI-PI score, which was reported by 5 responders in Sativex^®^ group, compared to 0.6 decrease in placebo group Co-consumption of additional medications
**Fallon et al., 2017 (study 1) [64]** Sativex oromucosal spray as adjunctive therapy in advanced cancer patients with chronic pain unalleviated by optimized opioid therapy: two double-blind, randomized, placebo-controlled phase 3 studies	
**Location**	United States (U.S.)
**Aim of study**	Assess efficacy (percent improvement in average daily pain NRS score) of Sativex^®^ as adjunctive analgesic in advanced cancer patients with pain unalleviated by optimized opioid treatment
**Study design**	RCT, phase III Double-blind, parallel group, multicenter N = 399 (n = 200 Sativex^®^ and n = 199 placebo) Patient population: Advanced cancer patients with incurable disease with average baseline NRS score 4–8 Study period: 2-week titration, followed by 3-week steady dose treatment Follow-up after 2 weeks of treatment termination
**Drug, administration, and dosing**	Sativex^®^ (Nabiximol) oromucosal spray Sativex^®^ dosing started with 1 spray/day, titrated by 1 spray/day (until max. daily dosing of 10 sprays/day) Average dosing: 7.4 sprays/day vs. 6.3 sprays/day in Sativex^®^ and placebo group, respectively
**Primary outcomes in pain**	Relative improvement in daily average pain on NRS score from baseline was non-significant (*p* = 0.27), with 7.2% Sativex^®^ vs. 9.5% placebo
**Side effects/** **adverse effects**	Withdrawal due to adverse effects: Sativex^®^ n = 38 (19%) vs. placebo n = 29 (14.6%) Adverse effects among others: Somnolence (n = 24, 12.1%) and nausea (n = 19, 9.5%), comparable to placebo despite nausea
**Additional notes**	Sativex^®^ favored according to quality of life Advised to maintain stable background analgesic co-consumption Subgroup of U.S. patients <65 years: Statistic favorable effect of Sativex^®^ (*p* = 0.04)
**Fallon et al., 2017 (study 2) [64]** Sativex oromucosal spray as adjunctive therapy in advanced cancer patients with chronic pain unalleviated by optimized opioid therapy: two double-blind, randomized, placebo-controlled phase 3 studies	
**Location**	U.S.
**Aim of study**	Assess efficacy (mean change in average daily pain NRS score) of Sativex^®^ as adjunctive analgesic in advanced cancer patients with pain unalleviated by optimized opioid treatment
**Study design**	RCT, phase III Double-blind, multicenter Enrichment enrollment randomized withdrawal (EERW) design and study period: Sativex^®^ titration for 10 days (single-blind) followed by 4 days stable dosing. Patients who obtained at least 15% NRS score pain improvement were randomized to Sativex^®^ (n = 103) or placebo (n = 103) self-titrated treatment for 5 weeks. N = 406 in EERW study (n = 78 did not reach least 15% pain improvement) Follow-up after 2 weeks of treatment termination
**Drug, administration, and dosing**	Sativex^®^ (Nabiximol) oromucosal spray Average dosing: 6.4 sprays/day Sativex^®^ during 2nd week of single-blind titration, with 6.5 sprays/day during double-blind period (vs. 6.3 sprays/day in placebo group)
**Primary outcomes in pain**	Mean change in average daily pain NRS score from Sativex^®^ treatment effect was non-significant (*p* = 0.92) compared to placebo n = 78/406 failed to obtain a 15% improvement in pain during the titration period
**Side effects/** **adverse effects**	Withdrawal due to adverse effects in EERW phase: Sativex^®^ n = 71 (17.5%) Adverse effects: Somnolence (n = 6, 5.8%) associated with Sativex^®^ group, but dizziness and nausea comparable to placebo group
**Additional notes**	Advised to maintain stable background analgesic co-consumption Sativex^®^ favored according to quality of life During treatment with Sativex^®^, mean change in average daily pain NRS score increased from 3.2–3.7, with analogues increase in placebo group (3.1–3.6), indicating a worsening in pain severity, which was speculated to be caused by high withdrawal rates
**Lichtman et al., 2018 [65]** Results of a double-blind, randomized, placebo-controlled study of nabiximols oromucosal spray as an adjunctive therapy in advanced cancer patients with chronic uncontrolled pain	
**Location**	U.S.
**Aim of study**	Assess efficacy (percent improvement in average pain NRS score) of Sativex^®^ as adjunctive analgesic in advanced cancer patients with uncontrolled pain by optimized opioid treatment
**Study design**	RCT, phase III Double-blind, multicenter N = 397 (n = 199 Sativex^®^ and n = 198 placebo) Patient population: Advanced cancer patients with NRS score 4–8 Study period: 2-week titration, followed by 3-week stable dosing treatment
**Drug, administration, and dosing**	Sativex^®^ (Nabiximol) oromucosal spray Sativex^®^ dosing started with 1 spray/day, titrated by 1 spray/day (until max. daily dosing of 10 sprays/day) Average dosing: 3.7 sprays/day Sativex^®^ during week 1 titration vs. 3.8 sprays/day placebo AND 7.3 vs. 6.4 sprays/day in Sativex^®^ vs. placebo, respectively, during last 4 weeks
**Primary outcomes in pain**	Improvement in median percent pain improvement on NRS score from baseline was non-significant (*p* = 0.09), with 10.7% Sativex^®^ vs. 4.5% placebo in intention-to-treat population
**Side effects/** **adverse effects**	Withdrawal due to adverse effects: Sativex^®^ n = 40 (20.1%) vs. placebo n = 35 (17.7%), which was due to the cancer disease or nausea
**Additional notes**	Tendency in favor of Sativex^®^ treatment in per-protocol population (*p* = 0.04) Stable dosing of background co-consumed analgesics advised Sativex^®^ might be beneficial in advanced cancer patients receiving lower doses of opioids due to, e.g., intolerance Beneficial effects of Sativex^®^ on secondary endpoints particularly in a U.S. subgroup population
